# Genital Warts in Women Vaccinated against HPV in Childhood: A Systematic Review

**DOI:** 10.3390/vaccines12050548

**Published:** 2024-05-17

**Authors:** Renata Malheiro, César Magalhães, Cláudia Camila Dias, Acácio Gonçalves Rodrigues, Carmen Lisboa

**Affiliations:** 1 Division of Microbiology, Department of Pathology, Faculty of Medicine, University of Porto, 4200-319 Porto, Portugal; up201807521@g.uporto.pt (R.M.); agr@med.up.pt (A.G.R.); 2 Department of Dermatology and Venereology, Gaia and Espinho Local Health Unit, 4434-502 Porto, Portugal; cesar.magalhaes@ulsge.min-saude.pt; 3 Centre for Health Technology and Services Research/Health Research Network (CINTESIS@RISE), Faculty of Medicine, University of Porto, 4200-319 Porto, Portugal; camila@med.up.pt; 4 Department of Community Medicine, Information and Health Decision Sciences (MEDCIDS) and Knowledge Management Unit, Faculty of Medicine, University of Porto, 4200-319 Porto, Portugal; 5 Department of Dermatology and Venereology, University Hospital Center of São João, 4200-319 Porto, Portugal

**Keywords:** human papillomavirus, anogenital warts, women, HPV vaccination, childhood, sexually transmitted infections, genital cancer

## Abstract

Human papillomavirus (HPV) is the most prevalent sexually transmitted infection among young women. Notably, more than ten years after the introduction of HPV vaccination programs in Europe, it is essential to review the real-world evidence of the incidence of anogenital warts (GWs) among women vaccinated during childhood. In this systematic review, three databases were searched for studies published between January 2008 and September 2023. Nine cohort studies were included. A total of 890,320 HPV-vaccinated women and 1,922,033 unvaccinated women were evaluated. All the studies but one investigated the 4vHPV vaccine. The incidence rate of GWs in vaccinated women ranged from 0.0 to 1650 per 100,000 person-years. The highest incidence rates were found in women vaccinated with one dose at the age of 17–19 years old and in fully vaccinated women only after 19 years of age. Similar incidence values were reported among unvaccinated women. The incidence of GWs was lower when the age at first dose was 9–11 years old. This systematic review reveals that the incidence of GWs among HPV-vaccinated women is related to the age of vaccination and the number of vaccine doses received. In the post-vaccination era, epidemiological surveillance of the incidence of GWs and their genotypes is crucial.

## 1. Introduction

Human papillomaviruses (HPVs) are DNA viruses responsible for the infection of mucosal and cutaneous epithelia in humans [1]. HPV is recognized as the most prevalent sexually transmitted disease among young women aged 18–25 years old [2]. GWs account for a significant portion of sexually transmitted diseases and are characterized by benign epidermal growth caused by HPV. They are more common in individuals with impaired immune systems but can also develop in those with adequate immune function [2]. Approximately 96–100% of all anogenital warts (GWs) include low-risk HPV types, namely types 6 and 11. High-risk HPV types, such as HPV 16, 18, 31, 33, and 35, can be identified in GWs [3]. GWs may harbor foci of intraepithelial neoplasia [4]. Notably, 30% of GWs will disappear within four months of their initial manifestations, yet most will reoccur within three months of completion of initial therapy, even if therapy is followed correctly [1]. Over the past decade, female HPV vaccination has proven effective in preventing HPV-related diseases such as cervical cancer, pre-malignant lesions, and GWs. In Europe, HPV vaccination has been gradually introduced in national immunization programs since 2007, with varying vaccine coverage across countries. Regions with high vaccine coverage target females between 10 and 13 years old, while areas with low coverage usually set the target between 11 and 15 years of age [5]. There are three prophylactic HPV vaccines available, namely, bivalent (2v), quadrivalent (4v), and nonavalent (9v) HPV vaccines, with a recommended schedule of a two-dose series administered 6–12 months apart and starting at the age of 9 years old [6]. The 2vHPV vaccine is only approved for girls, and the 4v and 9vHPV vaccines have been approved for both girls and boys [6]. According to the Advisory Committee on Immunization Practices, this vaccination can be extended to girls aged 13–26 years old and boys aged 13–21 years old who were not previously vaccinated [7]. The 2vHPV vaccine targets HPV 16 and 18, which cause approximately 70% of cervical cancers worldwide, while the 4vHPV vaccine targets HPV 16 and 18 and also protects against two low-risk HPV genotypes, HPV 6 and 11. The 9vHPV vaccine targets seven high-risk HPV genotypes (HPV-16/18/31/33/45/52/58) and two low-risk HPV genotypes, HPV 6 and 11 [8]. The overall (females and males combined) reported annual incidence of all GWs (including new and recurrent) ranged from 160 to 289 per 100,000, with a median of 194.5 per 100,000 [9]. Several studies have explored the effect of HPV vaccines on the prevention of infection and relapse of GWs among girls and women, including changes in the prevalence of HPV genotypes in GWs since the introduction of prophylactic vaccines. A study that aimed to assess the effectiveness of the 2v and 4vHPV vaccines in preventing GWs in young Spanish women found that the incidence of GWs in women who received the 2vHPV vaccine or unvaccinated women was higher than in those who received the 4vHPV vaccine [10]. Over the past decade, there has been a decline in HPV genotypes traditionally associated with GWs that were the primary targets of the first two prophylactic vaccines (2vHPV and 4vHPV), both in men and women. Although the prevalence of at least one of the four most common genotypes has remained stable, there has been a slight increase in infections involving multiple genotypes or at least one high-risk type [11]. Despite vaccination efforts, a gray area persists regarding data on changes in the paradigm of HPV types in GWs, highlighting the importance of this topic due to the high morbidity caused by HPV infection globally. GWs may affect sexual life, self-image, self-esteem, emotions, daily activities, and quality of life due to pain and discomfort, anxiety, and depression [12]. The impact of HPV vaccination on the epidemiology of GWs vaccination may be assessed after a short period of time following HPV. A systematic review of 10 years of real-world experience regarding the impact and effectiveness of the 4vHPV vaccine revealed a decline in the prevalence and incidence of GWs associated with a decrease in HPV6/11 infections [13]. Additionally, a changing trend for genotypes in the anogenital area of vaccinated women was reported [14]. More than a decade after the HPV vaccination programs, it is necessary to review real-world data regarding the incidence of GWs among vaccinated females. In the post-vaccination era, this epidemiological surveillance is crucial. We conducted a systematic review to answer the specific research question, what is the incidence of GWs in females vaccinated against HPV in childhood?

## 2. Materials and Methods

### 2.1. Search Strategy

This systematic review was performed in accordance with the standards and guidelines of the Preferred Reporting Items for Systematic Reviews and Meta-Analyses (PRISMA) 2020 checklist [15]. The PubMed, Scopus, and Web of Science databases were searched for studies published between 1 January 2008 and 1 September 2023, i.e., the period after the introduction of the HPV vaccine. The literature search was performed on 13 September 2023 and updated on 7 March 2024. The following combination of MeSH terms, title words, or abstract words included (“genital warts” or “anogenital warts” or “condyloma” or “condyloma accuminata”) AND (“girls” or “woman” or “children” or “childhood”) AND (“HPV vaccine” or “HPV vaccination”). Covidence Systematic Review Manager (Veritas Health Innovation, Melbourne, Australia) was used to upload the results from title and abstract screening. The titles and abstracts of the articles were reviewed by two independent and blinded reviewers (R.M. and C.L.) to determine potential eligibility. Full publications were reviewed for inclusion. Any conflicts were resolved through discussion with two other reviewers (C.M., A.G.R.). References from the included articles were searched for additional relevant literature. Details on the search strategy are reported in Supplementary Materials File S1.

### 2.2. Selection Criteria

Studies were eligible if they evaluated the incidence of GWs among women vaccinated against HPV in childhood and examined the same outcome in unvaccinated women. The criteria of exclusion were women vaccinated against HPV in adulthood with or without an HPV disease and studies reporting HPV-related diseases except GWs. Studies that included a solely male population and those that evaluated side effects, knowledge, barriers, cost-effectiveness, and immunogenicity related to HPV vaccines were also excluded. Additionally, we excluded ecological studies that analyzed the frequency of GWs in the pre-vaccination period and the post-vaccination period without the accurate identification of the vaccinated and unvaccinated female populations.

### 2.3. Data Extraction and Quality Assessment

Data were summarized from eligible publications using a standardized protocol constructed at the beginning of the literature search. Extracted information included the first author’s name and year of publication, study design, country or region where the study was conducted, the aim of the study, study population, methods/data source, vaccination program/coverage rate, type of HPV vaccine and definition of vaccinated women, follow-up, incidence rate of GWs per 100,000 person-years, conclusions, limitations, and quality assessment. Data were reviewed by the three researchers (R.M., C.L., C.M.). Furthermore, quality assessment was performed independently and blinded by these 3 researchers (R.M., C.L., C.M.) using the National Institutes of Health quality assessment tools [16]. Divergences were discussed between the researchers.

## 3. Results

**Figure 1:**
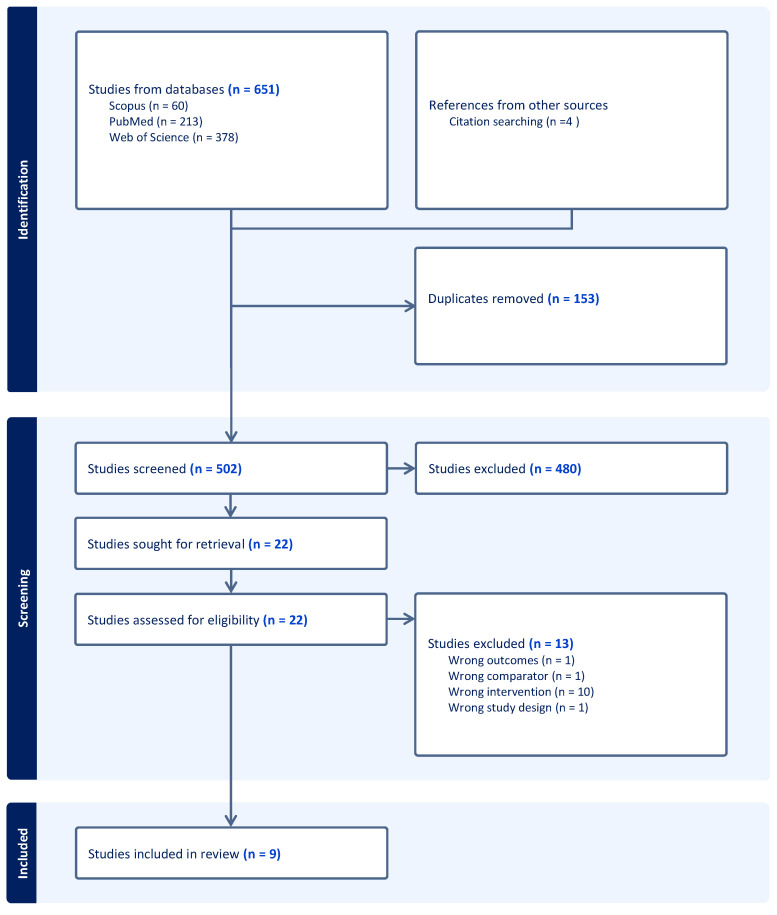
PRISMA flowchart.

A total of 651 articles were identified in our search, and after removing 153 duplicates, 498 were screened. According to our eligibility criteria, four studies were added after searching the citations of other papers. Out of 502 articles, 480 were excluded after analysis of the title and abstract. Then, 22 full-text articles were assessed for eligibility, and 13 were excluded because 10 evaluated an irrelevant intervention, 1 included an irrelevant population control (comparator), 1 did not use a study design that was considered eligible (review), and 1 reported an outcome not relevant to our research question (Figure 1).

### 3.1. Characteristics of the Included Studies

A total of nine studies were included in this systematic review: two prospective cohorts [17,18] and seven retrospective cohorts [10,17,18,19,20,21,22,23,24]. Seven studies were published between 2013 and 2018, and the remaining two studies were published in 2021 and 2022. Two studies [17,19] were conducted in Denmark; the remaining studies took place in Belgium, Spain (Valencia region), the USA, Canada (Manitoba region), Australia, the Netherlands, and Germany (Bavaria region) [10,20,21,23,24]. The main outcome assessed was the incidence rate of GWs in vaccinated women per 100,000 person-years in six studies [10,17,19,20,23,24]; three studies [18,21,22] presented the results as proportion of diagnosis of GWs. Table 1 shows the main characteristics of the included studies.

### 3.2. Study Population

This systematic review included nine cohorts that comprised data from a total of 2,812,353 women, 890,320 of whom had received an HPV vaccine. The study population comprised sexual health center attendees aged under 21 years in the study from Australia [21] and 16–24 years in the study from the Netherlands [18]. These studies included the lowest number of women vaccinated with at least one dose of HPV vaccine, 673 and 666, respectively [18,21]. Both cohorts from Denmark included female cohorts between 9–19 and 12–24 years old, with a vaccinated population of 248,403 and 485,408 women, respectively [17,19]. In Belgium, the target population involved older women (aged 19–54 years old), with a total study population of 106,579 and 43,399 vaccinated females [20]. A total of 185,973 privately insured adolescents from the USA were included in the study [23]. Three studies were conducted in regions. In Bavaria (Germany), the study population comprised women aged 9–28 years old, but only women aged 19–28 years old were considered for the analysis of the risk of GWs (n = 433,346) [22]. The study from Valencia (Spain) included girls aged 14–19 years old (n = 279,787) [10], and the one from Manitoba (Canada) included girls and women aged 9 years old and older (n = 125,791) [24].

### 3.3. Methods/Data Source

Six studies used data collected from population-based registries and health databases [10,17,18,19,21,24]. Other data sources, namely provider-sponsored health insurance plans such as Health MarketScan Commercial Claims, Reimbursement Database, and Statutory Health Insurance Physicians, were used in cohort studies conducted in the USA, Belgium, and Germany (Bavaria) [20,22,23]. Data concerning HPV vaccination were retrieved from these health registries in all studies, except for two [18,21] that collected self-reported vaccination status.

### 3.4. Type of Vaccination Program and Coverage Rate

While seven studies (7/9) [10,17,18,19,20,21,24] evaluated implemented national vaccination programs, one study (1/9) examined an implemented opportunist vaccination program [22], and the other (1/9) examined an HPV vaccine that was only available to privately insured adolescents [23]. The national vaccination programs of the included studies were implemented between 2007 and 2009. These school-based programs targeted girls aged 11 to 13 years old. The opportunist vaccination program targeted females aged 9–28 years old [22]. The American study targeted 9–18-year-old adolescents for HPV vaccination [8].

The coverage of at least one vaccine dose reached more than 70% in three studies [17,19,21]. Two studies reported one-dose coverage of 48% in Belgium [20] and 11% in Manitoba [24]. Moreover, coverage for three doses reached 68% in Manitoba [24], 44.9% in Valencia [10], and 40.9% in Bavaria [22].

### 3.5. Type of Vaccination

All the cohort studies examined the 4vHPV vaccine except the prospective cohort study by Woestenberg P. et al., which assessed the effect of the 2vHPV vaccine on GWs [18]. Moreover, the study by Osmani V. et al. included, up until 2016, girls vaccinated with the 4vHPV vaccine and a minority group of girls vaccinated with the 2vHPV vaccine; after 2016, the 9vHPV vaccine was used [22].

The control group was a group of unvaccinated women who had not received any dose of HPV vaccine in all the included studies, except for two [10,20], which also considered unvaccinated females if they had received a single dose of the 2vHPV vaccine.

### 3.6. Follow-Up

The study period of the analyzed cohorts varied between 2 years [21] and 12.42 years [24]. One study included in this systematic review analyzed the incidence of GWs from the beginning of vaccine implementation until 11 years later [17]. The study conducted in Manitoba, Canada, had a mean follow-up of 29 months for the analysis of the incidence of GWs, although the enrollment period was the longest, from 21 August 2001 to March 2013 [24].

### 3.7. Incidence of GWs

**Figure 2:**
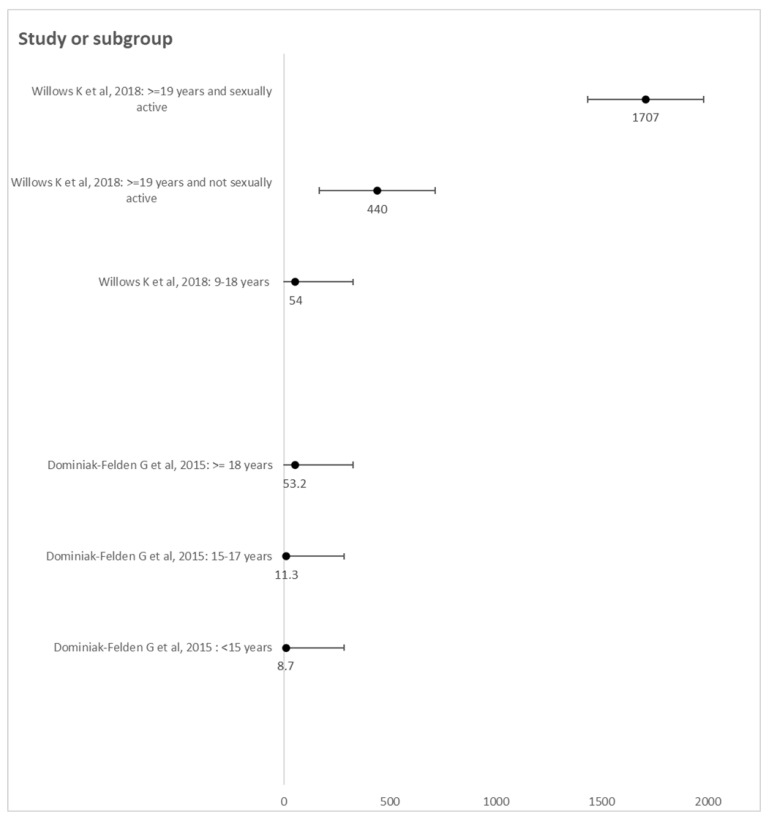
Incidence of GWs in young vaccinated women stratified by age of vaccination. Data from Willows K. et al. [24] and Dominiak-Felden G. et al. [20]. Note: IR 8.7 (IC 95% 3.6–20.9); IR 11.3 (IC 95% 5.1–25.1); IR 53.2 (IC 95% 17.2–165). IR 54 (IC 95% 38–75); IR 440 (IC95% 220–879); IR 1707 (IC 95% 1441–2022).

The incidence rate of GWs in women vaccinated reported by six cohorts varied from 0.0 to 1650 per 100,000 person-years. These values were reported in the same study [19]. The highest incidence rates of GWs were found in women vaccinated at 17–18 years old with less than two doses (>1000 per 100,000 person-years) and in 19-year-old girls with one dose (1650 per 100,000 person-years); such a rate of incidence was similar among unvaccinated girls/women [19]. Moreover, among women vaccinated at 19 years or older, whether sexually active or not, the incidence of GWs was found to be similar to that of unvaccinated women, regardless of the number of doses administrated, in the retrospective study conducted in Manitoba, Canada [24]. Regarding the age of vaccination, it ranged from 9–11 years old to over 19 years old. This incidence was 0.0 per 100,000 person-years in Danish girls vaccinated at the age of 9–11 years old and increased to 87.5 per 100,000 person-years at a vaccination age of 16–17 years old [17]. On the other hand, Baandrup et al. reported an incidence of less than 250 per 100,000 person-years in all dose groups among Danish girls vaccinated under 16 years old [19]. Concerning GWs in young vaccinated women stratified by age of vaccination, an incidence of 8.7 in girls fully vaccinated with the first dose at under 15 years old and an incidence of 11.3 if vaccination occurred at 15–17 years old were reported in the Belgian study [20]. On the other hand, an incidence of 54 per 100,000 person-years was also found in girls vaccinated at 9–18 years old in the study conducted in Manitoba, Canada [24] (Figure 2). Data reported by Blomberg et al. [17] and Baandrup et al. [19] were not presented due to the absence of confidence intervals, although the results were also stratified by age of vaccination.

**Figure 3:**
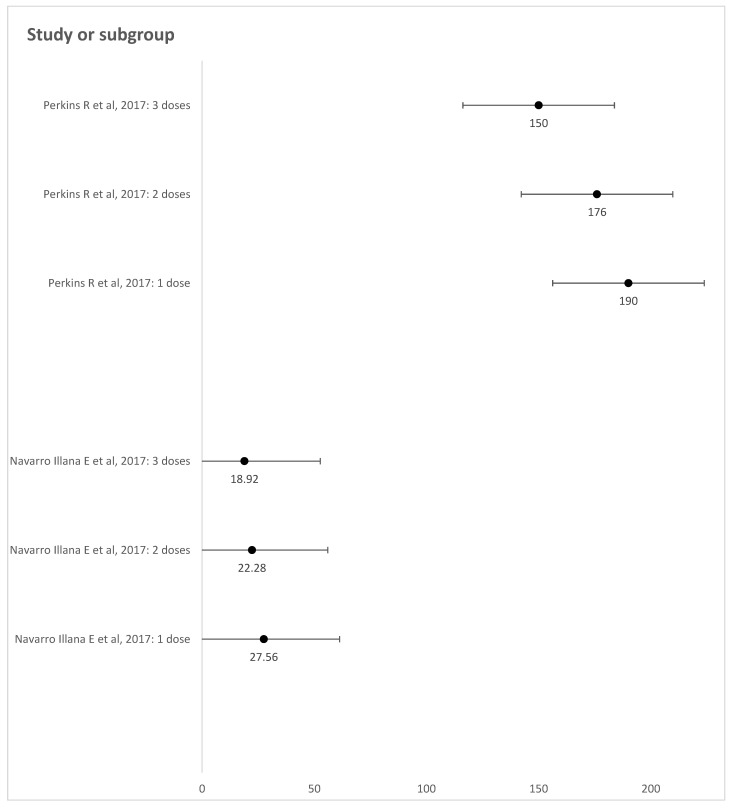
Incidence of GWs in young, vaccinated women stratified by the number of doses. Note: IR 27.56 (IC 95% 8.95–64.32); IR 22.28 (IC 95% 8.96–45.9); IR 18.92 (IC 95% 12.67–21.17) [10]. IR 190 (IC 95% 168–216); IR 176 (IC 95% 157–199); IR 150 (IC 95% 140–161) [23].

Two studies analyzed the incidence of GWs in young vaccinated women stratified by the number of doses [10,23] (Figure 3). Perkins et al. [23] compared the protection afforded by zero, one, two, and three doses of the 4vHPV vaccine against GWs. Among girls aged 9 to 18 years old, the incidence of GWs was 217 and 190 per 100,000 person-years among unvaccinated and one-dose-vaccinated girls, respectively. The incidence rate decreased to 176 and 150 per 100,000 person-years in girls vaccinated with two and three doses, respectively, as observed in Valencia [10]. In Valencia, the incidence rates were 27.56, 22.28, and 18.92 per 100,000 person-years for girls aged 12–13 years old vaccinated with one, two, and three doses, respectively. Additionally, the study from Manitoba [24] reported that among girls vaccinated at ages 9–18 years old, the incidence of GWs was 146, 153, and 39 per 100,000 person-years for those who received one, two, or three doses of the 4vHPV vaccine, respectively. Four studies [17,18,21,22] did not assess the incidence of GWs in vaccinated girls/women in relation to the number of doses of the HPV vaccine.

Regarding the two studies that reported proportion values of GWs, the prevalence ranged between 0% [21] and 0.9% [18] among women vaccinated at an age under 19 years old. For the 14–15-year-old group, the proportion with a diagnosis of GWs was 3.25% in vaccinated girls, and this proportion decreased to 0.52% and 0% for girls aged 15–16 and 16–17 years old [21]. On the other hand, Woestenberg et al. found a prevalence of GWs of 0.9%, both in partially and fully vaccinated girls with the 2vHPV vaccine at 13–18 years old. Among unvaccinated girls, this prevalence was 1.3% [18]. A third study [22] reported an incidence proportion of GWs ranging from 0.21% to 1.04% among 19–28-year-old women.

### 3.8. Quality Assessment (Figure 4)

A total of four studies included in this review were considered to be of good quality, three studies were considered fair, and two studies were considered to be poor according to the NIH tools [16]. The studies conducted in Australia [15] and the Netherlands [16] were considered to be of poor quality and scored negatively because of the small study population size, selection bias concerning the enrollment of STI attendees, and recall bias as information about HPV vaccination was self-recorded. Both studies assessed the prevalence of GWs rather than incidence. Additionally, there was a short period between exposure (HPV vaccination) and outcome (presence of GWs) to assess a causal relationship. The studies considered fair [10,20,22] have limitations concerning the implementation of exposure (vaccination), which was not consistent for all participants; there were also some potential confounding variables, such as GW diagnosis based on codification and status of HPV vaccination based on prescription data. The four studies [17,19,23,24] were considered to be good and scored positively concerning the definition of population and sample size, eligible rate, inclusion and exclusion criteria, the definition of exposure, and outcome. They assessed the efficacy of different doses of HPV vaccine, except for the prospective cohort study conducted in Denmark [17].

**Figure 4:**
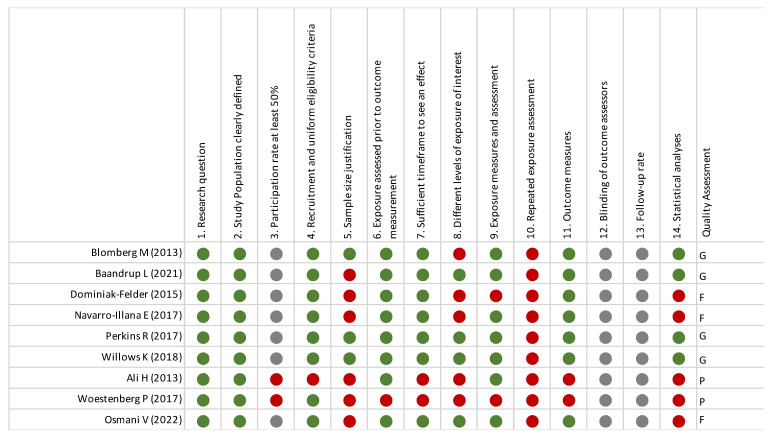
Quality assessment of the included studies [10,17,18,19,20,21,22,23,24].

The National Institutes of Health quality assessment tools for observational cohort and cross-sectional studies were used [16].

Quality was rated as P for poor (0–4 out of 14 questions), F for fair (5–8 out of 14 questions), or G for good (9–14 out of 14 questions). Green circle corresponds to 1 point, red circle to zero point and grey circle corresponds to Not Applicable or Not Reported.

## 4. Discussion

This systematic review shows that the incidence of GWs in women depends on the age of vaccination and the number of doses of HPV vaccine administered. The incidence rate of GWs is lower in vaccinated women compared to unvaccinated women, and this reduction is even higher in cases where vaccination occurs at younger ages. These findings are consistent with previous studies. However, women vaccinated against HPV are still at risk of developing GWs, and several factors might provide evidence of this. The studies included in this review aimed to assess the effect of HPV vaccination on the frequency of GWs by comparing vaccinated and unvaccinated women. Nonetheless, these studies are very heterogeneous in terms of sample size, age at enrollment and age at vaccination, clinical settings, definition of vaccination status, and follow-up, apart from differences between countries’ vaccination implementation programs. Furthermore, data concerning the age of vaccination proved to be difficult to obtain in some studies. In this systematic review, the overall incidence rate was stratified by age and number of doses of HPV vaccine. The incidence of GWs was lower in women vaccinated at younger ages. The prospective cohort published in 2013 found an incidence rate of GWs of 0.0 per 100,000 person-years among Danish girls vaccinated at 9–11 years old [17]. Nevertheless, the incidence estimate was performed after a short period following vaccination (3.1 years). No other study found such a value. Only two studies that evaluated the proportion of GW diagnoses described close but not comparable values [18,21]. The studies that assessed the incidence rate of GWs found an increase in incidence rate with age at vaccination, mainly among females vaccinated at over 15–16 years old and even more at over 19 years old. The study by Perkins et al. aimed to compare the protection afforded by the different 4vHPV vaccine doses against GWs. It concluded that one dose is associated with a higher frequency of GWs, and this frequency was similar to unvaccinated girls/women. However, the effectiveness of two and three doses was similar among a cohort of girls/women vaccinated between 9 and 18 years, but the risk of GWs increased with age [23]. In fact, the recommendation to vaccinate girls aged between 9 and 14 with two doses of the 4vHPV or 9vHPV vaccine and girls aged over 14 years old with three doses is supported by these findings [25]. Women vaccinated at age 19 years or older had a high incidence rate of GWs [17,19,20,24]. For one dose of the 4vHPV vaccine, the incidence rate of GWs ranges from 27.56 to 1650 per 100,000 person-years [10,19]. Data support the need for full vaccination with three doses, even though women maintain a high incidence in this age group [19,24]. Our systematic review includes data on various countries’ vaccine coverage rates. HPV vaccine coverage rate differs greatly from country to country. However, these differences are not reflected in our data since we compared previously identified vaccinated women with unvaccinated women belonging to the same age group. However, the number of unvaccinated girls/women was invariably higher than that of vaccinated girls/women in all included studies. The impact of HPV vaccination programs on the prevalence of non-vaccine HPV genotype infections in community settings has been assessed in a few studies. A study conducted in Switzerland [26] found a potential cross-protection effect of the 4vHPV vaccine using self-collected vaginal samples for HPV detection. This is consistent with a study conducted in the USA that found an 86% decline in the prevalence of 4vHPV vaccine genotypes among females aged 14–19 years, with the median age at first dose of 12 years [27]. It is also critical to check genotypes not targeted by the HPV vaccine. Li C et al. reported a prevalence of high-risk non-vaccine types of 25.4% and a prevalence of vaccine types of 9.3% [28] among females aged 18–35 who received the 4vHPV vaccine, based on self-collected cervicovaginal samples. In a recent study [14], it was noted that although HPV-51 is not included in the 9vHPV vaccine, its prevalence has been decreasing, along with HPV 31, 33, and 39 over the last decade in Taiwan. Regarding HPV genotypes in GWs, Freire-Salinas J. et al. [29] found a significant decrease in the prevalence of HPV6 and 11, which are responsible for the majority of GWs, following vaccination with the 2v and 4vHPV vaccines, in contrast to HPV 16 and 18. However, a significant increase was observed in HPV 31, 45, and 52. Additionally, another Spanish study **[**] reported that multiple HPV types in GWs increased from 14.2% to 26.6%. Furthermore, there has been an increase in genotypes not targeted by the vaccines, such as HPV-51, HPV-31, HPV-52, and HPV-73, after the introduction of HPV vaccines. Our review has several limitations. There are several limitations to the studies included in this review. One limitation shared by many of the studies is the reliance on administrative data, which may lead to misclassification or non-codification, contributing to an underestimation of the incidence of GWs. Moreover, some studies exhibited participant selection bias, limiting the generalizability of the results and compromising their external validity. Additionally, the cohorts (vaccinated vs. unvaccinated and cohorts stratified by age and by number of vaccine doses) are not completely comparable, with differences in sample size, risk of HPV exposure, and follow-up time. The broad age range for HPV vaccination further complicates the identification of vaccinated individuals during childhood. A limitation of our review arises from our decision to restrict the comparison of the outcome (genital warts) to vaccinated women vs. unvaccinated women. This approach led to the exclusion of ecological studies that estimated the incidence rate of GWs in the pre-vaccination era and post-vaccination era. Another limitation may stem from our focus on the incidence of GWs in real-world/clinical settings, which led to the exclusion of clinical trials and limited knowledge about HPV status prior to vaccination. However, we followed this approach to consolidate real-world data regarding GWs and to assess the population of vaccinated females who develop GWs. Finally, the issue of changing HPV genotypes in GWs ten or more years after HPV vaccination warrants ongoing epidemiological surveillance.

## 5. Conclusions

This systematic review found that the incidence of GWs in women is highly related to the age at vaccination and the number of doses of the 4vHPV vaccine. Despite the limitations present in the included cohort studies, this review provides valuable insights into the post-HPV-vaccination epidemiology of GWs among females from different countries with diverse vaccination implementation schemes. The findings are consistent with previous studies in this area. However, our clinical data suggest that there is still a considerable risk of GWs, and several factors may contribute to this risk. Continued monitoring of HPV genotypes in GWs, including both vaccine-targeted and non-targeted types, is crucial to assess the potential for cross-protection or genotype selection and to guide effective public health interventions.

## Figures and Tables

**Table 1:** Main characteristics of the studies included in this review.

First Author, Publication Year	Study Design	Country/Region	Aim	Study Population	Methods/Data Source	Vaccination Program Coverage Rate	Type of Vaccination (Vaccinated Definition)	Study Period Follow-Up	Incidence Rates of GWs per 100,000 Person-Years	Conclusions	Limitations	Quality Assessment
Blomberg M. et al., 2013 [17]	Prospective cohort	Denmark	Assess the effect of HPV vaccination on risk of GWs	Girls in the birth cohorts 1989–1999/age of vaccination 9–19 years old (n total = 399,967; vaccinated = 248,403)	Population-based registries. National Health Insurance Service Register and Prescription Registry. Data on incident cases of GWs among vaccinated and unvaccinated girls.	National vaccination program in January 2009 for girls aged 12 and catch-up for girls aged 13–15 since October 2008. Coverage: between 87% and 90% for at least 1 dose.	4vHPV vaccine (vaccinated: at least 1 dose)	Vaccinated 3.1 years; unvaccinated 3.5 years	Vaccinated vs. unvaccinated according to age of vaccination: 9–11 years: 0.0 vs. 2.8 12–13 years: 3.0 vs. 5.5 14–15 years: 29.4 vs. 34.6 16–17 years: 87.5 vs. 264.7 18–19 years: 256.0 vs. 385.9	Highly significant reduction in the occurrence of GWs among vaccinated girls. No GWs occurred among girls vaccinated at 9–11 years old.	Self-selection bias in the oldest birth cohorts. Exclusion of prevalent infections. Diagnosis of GWs could only be made at hospitals and outpatient clinics (results may not be generalizable). Shorter follow-up for younger cohort.	Good
Baandrup L. et al., 2021 [19]	Retrospective cohort	Denmark	Assess the VE of 1 or 2 doses of HPV vaccination based on the risk of GWs and compare 1-dose VE with 2- or 3-dose scheme according to age at first dose and over time.	Females born in 1985–2003/Females aged from 12 to 24 years (n total = 1,076,945 Vaccinated = 485,408)	Civil Registration System; National Health Service Register; National Prescription Registry; Statistics Denmark.	Vaccination initiated in 2009 for 12-year-olds; a first catch-up for girls aged 13–15; a second catch-up for females aged up to 27 years old. Coverage: ≥75% for at least 1 dose.	4vHPV vaccine (vaccinated: at least 1 dose)	Up to 10 years	Unvaccinated: 19–20 y: 1617 Vaccinated: 12–14 years/15–16 years <250 and remained low during follow-up (in all dose groups) 17–18 years ≤2 doses: >1000 in the first year and then decreased 3 doses: 172 in the first year and remained low during follow-up ≥19 years 1 dose: ~1650 (similar to unvaccinated at age ≥ 19 y with 1 dose) ≥2 doses: >1000 first year, then dropped to ~250 afterwards.	1 or 2 doses of 4vHPV vaccine was associated with substantial protection against GWs in girls vaccinated at age ≤ 16 years	Bias related to girls who were sexually active before the vaccination. Contribution of 1 dose/person-time is limited and 1-dose estimate was influenced by diagnosis of GWs.	Good
Dominiak-Felden G. et al., 2015 [20]	Retrospective cohort	Belgium	Evaluate beneficial effects of the 4vHPV vaccine on GWs in Belgium	All women aged 16–59 years affiliated with MLOZ. (n total = 106,579; vaccinated= 43,399.)	Large sick-fund reimbursement database (MLOZ)	National vaccination program since December 2008 for all girls aged 12 to 18 years. Opportunist vaccination program in 2007 Coverage in 2013 for at least 1 dose: 48% for 16–22-year-olds 2% for 23–30-year-olds	4vHPV vaccine Fully vaccinated: 3 doses Partially vaccinated ≤ 2 doses Unvaccinated: no 4vHPV vaccine or any dose of 2vHPV vaccine	Jan 2006 to December 2013 (8 years)	Unvaccinated: 111.7 Fully vaccinated with first dose: <15 years: 8.7 15–17 years: 11.3 ≥18 years: 53.2 Partially vaccinated: 1 dose: 70.5 2 doses: 33.8	GW incidence rates decreased significantly for vaccinated women. This decrease was highest in girls vaccinated at a younger age.	Bias due to study design and database sources. Misclassification of status of HPV exposure: underestimation of vaccine effectiveness. Overestimation of incidence rates of GWs. A first GW episode was defined as an agreement for a first prescription of imiquimod.	Fair
Navarro-Illana E. et al., 2017 [10]	Retrospective cohort	Spain (Valencia)	Assess the effectiveness of 4v and 2vHPV vaccines in preventing GWs	All girls aged 14–19 years who were registered in the Valencian community (n total = 279,787)	Health databases	National HPV immunization program in 2008 indicated for girls aged 12–13 years old. Coverage of three doses of 4vHPV: 44.9% for girls aged 14 years old.	4vHPV vaccine 2008–2010; 2vHPV ≥ 2011 Vaccinated: at least 1 dose of 4vHPV vaccine. Unvaccinated: no 4vHPV and received 2vHPV vaccine (any dose).	January 2009 to December 2014	Unvaccinated 4vHPV: 94.07 4vHPV Vaccinated: 1 dose: 27.56 2 doses: 22.28 3 doses: 18.92	1. 4vHPV vaccine was effective against GWs in this population, even with low vaccine coverage. 2. There was a non-significant decrease in the risk of GWs in line with the number of doses of 4vHPV vaccine received. 3. Unvaccinated girls and those vaccinated with the 2vHPV vaccine had the same risk of incidence of GWs.	Bias relative to data collected. Underestimation of incidence of GWs (diagnosis may not have been codified—use of just one diagnosis code). Cohorts are not completely comparable—women who received 2vHPV vaccine had not reached the age at which the highest incidence of disease has been reported.	Fair
Perkins R. et al., 2017 [23]	Retrospective cohort	United States of America	Compare the relative protection afforded by 0, 1, 2, and 3 doses of 4vHPV vaccination against GWs	All girls aged 9–18 years old on 1/1/2007. Average age 16.3–16.9 years old; n total = 387,906; vaccinated=185,973).	Truven Health Analytics MarketScan Commercial Claims Database	Privately insured adolescents who could afford HPV vaccination	4vHPV vaccine 1, 2 doses < 5 or >= 5-month interval or 3 doses of 4v NR	1 January 2007–12 December 2013 (7 years) Average length of follow-up 5.64 years	Overall: 197 Unvaccinated: 217 Vaccinated: 1 dose—190 2 doses—176 3 doses—150	Receipt of 0 or 1 dose was associated with more GWs than 3 doses. The effectiveness of 2 doses was similar to 3 doses. The risk of GWs increased with age.	Short intervals between doses and small number of girls with GWs receiving 2 doses. Groups differ in their risk of HPV exposure Bias due to use of administrative data. No stratification by age of vaccination.	Good
Willows K. et al., 2018 [24]	Retrospective cohort	Canada (Manitoba)	Assess the effectiveness of 4vHPV vaccination program in Manitoba, Canada, in reducing incidence of GWs and to what extent effectiveness depends on age at vaccination and number of doses	All girl participants of 9 years and older who were registered in Manitoba Health’s vaccine registry. (n total = 125,791; vaccinated with at least 1 dose = 31,464.)	Population-based cohorts. Health’s vaccine registry, population registry, immunization monitoring system, and medical services databases.	School-based program for all girls aged 11–12 years old in September 2008; catch-up for girls aged 9–26 deemed at “high risk” for HPV infection (November 2012–2014). Coverage: 3 doses—68%; 2 doses—21%; 1 dose—11%.	4vHPV Vaccine	21 August 2001 to March 2013	Females aged 9–18 years Unvaccinated: 94 Vaccinated: 54 (1 dose: 146; 2 doses: 153; 3 doses: 39) ≥19 y and not sexually active Unvaccinated: 321 Vaccinated: 440 (1 dose: 860; 2 doses: NR; 3 doses: 449) ≥19 y and sexually active Unvaccinated: 546 Vaccinated: 1707 (1 dose: 2797; 2 doses: 2007; 3 doses: 1379)	Females vaccinated at age 18 years or younger were associated with a 40% reduction in GW risk. For women vaccinated at an older age, the risk of GWs remained increased regardless of the number of doses.	Underestimated the disease’s incidence rates. Clinical markers used to suggest prior sexual activity are not a very sensitive indicator of previous HPV exposure. Short follow-up time. Inability to calculate VE estimates for age groups or interactions between age groups and number of vaccine doses.	Good
Ali H. et al., 2013 [21] *	Retrospective cohort	Australia	Assess effect of the 4vHPV vaccination program on GWs in Australia 5 years after the program was established	Australian-born patients who attended the two largest clinics (Melbourne Sexual Health Centre and Sydney Sexual Health Centre) from 2009 to 2011. (n total = 834; vaccinated = 673.)	Data collected in the two STI clinics on patients’ demographics, behavior, clinical diagnosis of GWs, and self-reported HPV vaccination status of new patients from 2009 to 2011	Vaccination initiated in 2007 for girls aged 12–13 years in schools; since 2007–2009, catch-up for 13–18-year-old schoolgirls and 18–26-year-olds in the community. Coverage in 12–13-year-old girls: 1 dose—83%; 2 doses—80%; 3 doses—73%.	4vHPV vaccine	2009 to 2011	Proportion of GW * diagnosis in vaccinated vs. unvaccinated: 14–15 years: 3.25% (8/246) vs. 8.62% (5/58) 15–16 years: 0.52% (1/192) vs. 6.25% (3/48) 16–17 years: 0% (0/235) vs. 7.27% (4/55)	The proportion of GWs in girls vaccinated at under 17 years old is lower than in unvaccinated girls in the same age group	Selection bias: sexual health services target populations that are at higher risk of STIs, so they are expected to have a higher incidence of GWs than in the general population. Self-reported HPV vaccination. No record of date of vaccination or number of doses of vaccine. Low number of participants. No data on incidence rate.	Poor
Woestenberg P. et al., 2017 [18] *	Prospective cohort	The Netherlands	Assess the effects of the 2vHPV-16/18 vaccine on genital HPV-6 and/or HPV-11 positivity and GWs by comparing vaccinated and unvaccinated women with similar exposure	Females aged 16 to 24 years old who attend STI clinics. (n total= 1198.)	National surveillance database. Data collected from PASSYON: Papillomavirus Surveillance among STI clinics. It included genital self-swab and questionnaire comprising self-reported vaccination status.	National vaccination program since 2010 for girls aged 13 years old. Opportunistic vaccination program in 2009 for girls aged 12–16 years old. Coverage in 2010: 56% for 13-year-olds and 52% for catch-up cohorts.	2vHPV vaccine. Fully vaccinated: 3 doses.	2011–2015	Prevalence of GWs * (in girls vaccinated at 13–18 years old): Unvaccinated: 1.3% (6/447) Vaccinated at least once: 0.9% (6/665) Fully vaccinated: 0.9% (4/466)	1. No cross-protective effect of the 2vHPV vaccine on genital HPV-6/11 positivity. 2. Non-significant partially protective effect on GWs.	Low number of GW diagnoses. Recall bias. Differences between vaccinated and unvaccinated women. Selection bias: sexual health services typically target populations that are at higher risk of STIs and were vaccinated primarily with the catch-up vaccination. 2vHPv vaccine does not provide cross-protective effectiveness against HPV6/11. No data on incidence rate.	Poor
Osmani V. et al., 2022 [22] *	Retrospective cohort	Germany (Bavaria)	Investigate the effects of HPV vaccination on the risk of GWs and precancerous cervical lesions in vaccinated and unvaccinated young women considering the vaccine type and contraceptive prescription prior to vaccination	Females born between 1990 and 2009. Age range for vaccination 9 to 28 years old. (n total = 433 346 women aged 19 to 28 years old for analysis of the risk of GWs.)	Bavarian Association of Statutory Health Insurance Physicians (KVB).	Opportunistic vaccination for females aged 9 to 28 years old. Coverage in 2018 for fully vaccinated women: 40.9% of 18-year-olds 13.3% of 12-year-olds	4vHPV vaccine: mostly until 2016; 9vHPV vaccine: mostly after 2016; 2vHPV vaccine declines from 13.9% in 2011 to 1.3% in 2018. Partially vaccinated: 1 or 2 doses according to age. Unvaccinated: any dose.	8 years (2011 to 2018)	Not/partially/fully vaccinated (%): * - 19 years: 0.44/0.20/0.21 - 20 years: 0.72/0.35/0.30 - 21 years: 1.06/0.58/0.49 - 22 years: 1.46/0.75/0.62 - 23 years: 1.90/0.68/0.61 - 24 years: 2.14/0.75/0.81	Vaccinated compared to unvaccinated women had a lower incidence of GWs; however, only small differences were detected between fully and partially vaccinated women, and these findings were independent from age.	Underestimated incidence of GWs: diagnoses are based on ICD-10 and females who did not visit an office-based physician were not diagnosed. Analyses of the outcome based on the information on HPV vaccination in the drug prescription data. Information about potential confounders was not available. Bias due to assuming that prescription of hormonal contraceptives was an indicator of sexual activity. Bias due to 2vHPV vaccine. No data on incidence rate.	Fair

Notes: * Proportion of diagnosis of GWs Ali H. et al., 2013 [21]; Woestenberg P. et al., 2017 [18]; and Osmani V. et al., 2022 [22]. Abbreviations: NR, not reported; GWs, anogenital warts; STIs, sexually transmitted infections; VE, vaccine effectiveness; 4v, quadrivalent HPV vaccine; 2v, bivalent HPV vaccine; 9v, nonavalent vaccine.

## Data Availability

Data are contained within the article and Supplementary Materials.

## Supplementary Materials

The following supporting information can be downloaded at: https://www.mdpi.com/article/10.3390/vaccines12050548/s1, Supplementary Material S1. Search strategy and study selection criteria.
