# Supplementary material for: Genital Warts in Women Vaccinated against HPV in Childhood: A Systematic Review
Source: Vaccines (Basel). 2024 May 17;12(5):548. doi: 10.3390/vaccines12050548 (PMC11126080; doi:10.3390/vaccines12050548)
Supplement: Supplementary file 1 [file vaccines-12-00548-s001.zip › vaccines-2955304-supplementary.docx]

Systematic Review

Genital warts in the post-HPV vaccination era: An eradicated condition among young women? A systematic review

Renata Malheiro^1^, César Magalhães^2^, Acácio Gonçalves Rodrigues^,1,3^, Carmen Lisboa^1,3,4^ *

| **Citation:** To be added by editorial staff during production.  Academic Editor: Firstname Lastname  Received: date  Revised: date  Accepted: date  Published: date  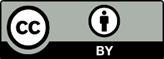  **Copyright:** © 2024 by the authors. Submitted for possible open access publication under the terms and conditions of the Creative Commons Attribution (CC BY) license (https://creativecommons.org/licenses/by/4.0/). |
| --- |

^1^Division of Microbiology, Department of Pathology, Faculty of Medicine, University of Porto, 4200-319 Porto, Portugal.

^2^Department of Dermatology and Venereology, ULS Gaia/Espinho, 4434-502 Porto, Portugal.

^3^Centre for Health Technology and Services Research/Health Research Network (CINTESIS@RISE), Faculty of Medicine, University of Porto, 4200-319 Porto, Portugal.

^4^Department of Dermatology and Venereology, ULS São João, 4200-319 Porto, Portugal.

*Correspondence: carlis@med.up.pt

**Supplementary Material S1**. Search strategy and study selection criteria

Search strategy : Original search- 651 results, searched on 13/10/2023 and updated 7/03/2024

| MEDLINE (Pubmed)- 213 results  ((((((((((((((((((((((genital warts[Title/Abstract])) OR (anogenital warts[Title/Abstract])) OR (condyloma accuminata[Title/Abstract])) OR (condyloma[Title/Abstract])) OR (hpv disease[Title/Abstract])) AND (girls[Title/Abstract])) OR (woman[Title/Abstract])) OR (women[Title/Abstract])) OR (young woman[Title/Abstract])) OR (young women[Title/Abstract])) OR (childhood[Title/Abstract])) OR (children[Title/Abstract])) AND (vaccine[Title/Abstract])) OR (vaccinated[Title/Abstract])) OR (vaccination[Title/Abstract])) OR (hpv vaccination[Title/Abstract])) OR (hpv non-vaccine type[Title/Abstract])AND (NOT (review[Publication Type])) NOT (editorial[Publication Type])) NOT (attitudes[Title/Abstract])) NOT (knowledge[Title/Abstract])) NOT ("barriers"[Title/Abstract])) NOT (awareness[Title/Abstract])) NOT (perceptions[Title/Abstract])) NOT (acceptability[Title/Abstract])) NOT ("cost effectiveness"[Title/Abstract])) NOT (opportunities[Title/Abstract])) NOT ("immunogenicity"[Title/Abstract]))) NOT (efficacy[Title/Abstract])) NOT (effectiveness[Title/Abstract])) ) AND (hpv monitoring[Title/Abstract]))) OR (hpv post-vaccination[Title/Abstract])) OR (hpv post-vaccinated[Title/Abstract]). Filters from 2008 -2023  Scopus – 60 results  (TITLE-ABS(genital warts) OR TITLE-ABS(anogenital warts) OR TITLE-ABS(condyloma accuminata) OR TITLE-ABS(condyloma) OR TITLE-ABS(hpv disease) AND TITLE-ABS(girls) OR TITLE-ABS(woman) OR TITLE-ABS(women) OR TITLE-ABS(young woman) OR TITLE-ABS(young women) OR TITLE-ABS(childhood) OR TITLE-ABS(children) AND TITLE-ABS(vaccine) OR TITLE-ABS(vaccinated) OR TITLE-ABS(vaccination) OR TITLE-ABS(hpv vaccination) OR TITLE-ABS(hpv non-vaccine type) AND NOT DOCTYPE(review) AND NOT DOCTYPE(editorial) AND NOT TITLE-ABS(attitudes) AND NOT TITLE-ABS(knowledge) AND NOT TITLE-ABS(barriers) AND NOT TITLE-ABS(awareness) AND NOT TITLE-ABS(perceptions) AND NOT TITLE-ABS(acceptability) AND NOT TITLE-ABS(cost effectiveness) AND NOT TITLE-ABS(opportunities) AND NOT TITLE-ABS(immunogenicity) AND NOT TITLE-ABS(efficacy) AND NOT TITLE-ABS(effectiveness)) AND (TITLE-ABS(hpv monitoring) OR TITLE-ABS(hpv post-vaccination) OR TITLE-ABS(hpv post-vaccinated)) AND PUBYEAR > 2007 AND PUBYEAR < 2024 |
| --- |
| Web of Science- 378 results  ((((((((((((((((((((((((((((((((TS=(genital warts)) OR TS=(anogenital warts)) OR TS=(condyloma acuminata)) OR TS=(condyloma)) OR TS=(hpv disease)) AND TS=(girls)) OR TS=(woman)) OR TS=(women)) OR TS=(young woman)) OR TS=(young women)) OR TS=(childhood)) OR TS=(children)) AND TS=(vaccine)) OR TS=(vaccinated)) OR TS=(vaccination))) OR TS=(hpv vaccination)) OR TS=(hpv non-vaccine type)) NOT DT=(Review OR Editorial Material)) NOT TS=(attitudes)) NOT TS=(knowledge)) NOT TS=(barriers)) NOT TS=(awareness)) NOT TS=(perceptions)) NOT TS=(acceptability)) NOT TS=(cost effectiveness)) NOT TS=(opportunities)) NOT TS=(immunogenicity)) NOT TS=(efficacy)) NOT TS=(effectiveness)) AND TS=(hpv monitoring)) OR TS=(hpv post-vaccination )) OR TS=(hpv post-vaccinated) AND 2008 OR 2009 OR 2010 OR 2011 OR 2012 OR 2013 OR 2014 OR 2015 OR 2016 OR 2017 OR 2018 OR 2019 OR 2020 OR 2020 OR 2021 OR 2022 OR 2023 |

Citation Search: 4 results added from other articles
